# Pericarditis in Lupus

**DOI:** 10.7759/cureus.4166

**Published:** 2019-03-01

**Authors:** Eric Dein, Homeyra Douglas, Michelle Petri, Genevieve Law, Homa Timlin

**Affiliations:** 1 Rheumatology, Johns Hopkins Bayview Medical Center, Baltimore, USA; 2 Cardiology, Aintree University Hospital, Liverpool, GBR; 3 Rheumatology, The Johns Hopkins University School of Medicine, Baltimore, USA; 4 Rheumatology, FETCH (For Everything That's Community Health) South Island, Victoria, CAN; 5 Internal Medicine, The Johns Hopkins University School of Medicine, Baltimore, USA

**Keywords:** systemic lupus erythematosus, pericarditis

## Abstract

Pericarditis is a common cardiac manifestation in systemic lupus erythematosus (SLE). Serositis is recognized in the ACR, SLICC, and EULAR/ACR classification criteria. We reviewed the prior research regarding the epidemiology, risk factors, presentation, and treatment of pericarditis in SLE.

## Introduction and background

Systemic lupus erythematosus (SLE) is a multisystem disease with a variety of clinical presentations and manifestations. The ACR, SLICC, and EULAR/ACR criteria all include serositis as a clinical classification [1-3]. Serositis can manifest as pleuritis, pericarditis, or peritonitis [4]. We reviewed the literature regarding the presentation and treatment of pericarditis and myocarditis in SLE.

## Review

Epidemiology

Serositis is a common manifestation of SLE. In a 10-year and 1,000 patient cohort of SLE, Cervera noted 16% of SLE patients had serositis, including pleuritis and/or pericarditis [5]. The incidence of pericarditis has widely ranged in the literature between 11-54% [4, 6-8]. Part of the variation in reported data is the difference between symptomatic and asymptomatic pericardial involvement. In studies of symptomatic pericarditis through routine clinical diagnoses, the incidence is reported to be about 25%, though asymptomatic pericardial findings have been seen on echocardiograms in more than 50% of SLE patients [9].

Risk factors

A study of 2,390 SLE patients by Ryu et al. identified the risk factors for pericarditis, as well as pleurisy. They found that hemolytic anemia, proteinuria, lymphadenopathy, and anti-Smith (anti-Sm) antibodies were associated with pericarditis, whereas pulmonary fibrosis and gastrointestinal infarction were only associated with pleurisy. Fever, Raynaud’s phenomenon and anti-DNA were associated with both pericarditis and pleurisy [6]. Male gender and younger age at diagnosis have been associated with elevated risk of serositis and pericarditis [10-11]. Zhao et al. reported that serositis was more commonly seen with lupus nephritis, interstitial lung disease, pulmonary arterial hypertension, leukopenia, thrombocytopenia, and hypocomplementemia [8]. Szodoray et al. found that a low level of vitamin D was associated with pericarditis in SLE [12]. In addition to the association with anti-Sm antibodies [6, 11, 13-15], other research has linked anti-Jo1 [13], anti-double stranded DNA (anti-dsDNA) [8, 15], and anti-ribonucleoprotein (anti-RNP) [11,15] to serositis/pericarditis. Jurencak studied pediatric SLE patients and found a predisposition to serositis with antibodies to anti-chromatin anti-ribosomal P and anti-La, in addition to anti-RNP, anti-Sm, anti-dsDNA and anti-Ro [15].

Histology/pathophysiology

The inflammation of the pericardium and myocardium is mediated by immune complexes [7, 16]. Direct immunofluorescence revealed fine granular immune complexes and the deposition of complement within the perivascular blood vessels [7]. Acute pericarditis is characterized by serofibrinous or fibrinous changes to the pericardium, which becomes fibrous or fibrofibrinous in chronic pericarditis [16]. Vascular proliferation is demonstrated on histology in patients with acute pericarditis [17]. The pericardial fluid within an effusion reveals exudative findings [17]. Patients who develop cardiac tamponade as a result of effusion have a statistically significant lower level of complement 4 (C4), compared to patients without tamponade, further suggesting an immune-complex and complement-mediated pathway in serositis [18].

Clinical findings and diagnosis of pericarditis

The presence of pericarditis patients with SLE is similar to the classic presentations of acute pericarditis, with typical precordial or substernal pleuritic chest pain with positional variation in pain (relieved by sitting upright) [7]. Patients may have dyspnea, fever, tachycardia, and decreased or muffled heart sounds. Physical examination may reveal a pericardial rub, but its absence does not exclude the diagnosis [7]. Electrocardiogram classically reveals diffusely elevated ST segments with peaked t-waves, though more commonly shows non-specific t-wave changes or transient ST changes are noted [7].

Recurrence after the first episode of pericarditis is not uncommon and is seen in 15-30% of cases of acute pericarditis [19]. Pericarditis can also occur in Whipple's Disease, Behcet's Disease, Inflammatory Bowel Disease, IgG4-related disease versus cardiac sarcoidosis versus idiopathic recurrent acute pericarditis (IRAP).

Pericarditis may occur as a manifestation of Whipple’s disease, even in the absence of gastrointestinal symptoms. Pericarditis is the most common cardiac manifestation of inflammatory bowel disease and has been reported to occur in approximately 70% of cases with cardiac involvement [20].

Pericarditis as a presenting feature of IgG4-related disease, often with a hemodynamically significant pericardial effusion resulting in tamponade, has been increasingly recognized and reported by a number of investigators in recent years [21]. The pericardial fluid in at least one of these cases was reported to be associated with elevated interleukin-10 (IL-10) and adenosine deaminase levels [22]. The diagnosis in all of these cases, however, requires a pericardial biopsy with consistent histopathologic features. The 2011 International Consensus Statement on the pathology of IgG4-related disease states that the presence of at least 2 of the 3 major histopathologic criteria, which are obliterative phlebitis, storiform fibrosis and the presence of a dense lymphoplasmacytic infiltrate in the specimen, are required for diagnosis [23]. Peripheral eosinophilia and elevated serum IgG4 levels, while suggestive, are neither diagnostic nor be present in patients with IgG4-related disease [24].

Isolated cardiac sarcoidosis without clinically apparent disease in other organ systems has been described [25]. Pericarditis and pericardial effusions as complications of cardiac sarcoidosis have been documented as case reports in the literature [26]. However, arrhythmias and acute heart failure from myocarditis are much more common presentations of cardiac sarcoidosis [27].

IRAP, a diagnosis of exclusion, is estimated to occur as a long-term complication for up to 20-50% of cases of acute pericarditis [28]. Whether or not the diagnosis is driven by an autoimmune versus an auto-inflammatory derangement is unclear. Familial clustering has been recently reported in up to 10% of patients with recurrent pericarditis, suggesting a genetic predisposition for at least some cases of IRAP [29]. Subsequent reports have suggested that up to 6% of IRAP cases are associated with mutations in the TNFRSF1A gene encoding the 55-kD receptor for tumor necrosis factor-α [28]. Such cases have been reported to respond clinically to anakinra, an interleukin-1 (IL-1) inhibitor [30].

All of the aforementioned differential considerations require a pericardial biopsy to establish a diagnosis. Surgical pericardial stripping is a therapeutic option for recurrent tamponade.

Cardiac tamponade is a critical diagnosis to consider, though is an uncommon presentation in SLE. Approximately 2% of patients will develop cardiac tamponade or constrictive pericarditis [9, 17]. Radiographs are only useful for large effusions and show an enlarged cardiac silhouette or “water bottle sign”. The standard imaging modality for pericardial effusions is transthoracic echocardiogram, which can show mild effusions and thickening of pericardium [9]. Kahl et al. found that the most common initial finding of tamponade in SLE was venous congestion, presenting as ascites, facial or peripheral edema [17]. The size of effusion on echocardiogram is the most powerful predictor of patient outcome in pericardial effusions, whereas right-sided chamber collapse and/or inferior vena cava plethora/response to respiration did not add additional prognostic value [31].

Other considerations in the evaluation

Alternative diagnoses should be carefully considered in the evaluation of patients with signs or symptoms of pericarditis. Other causes of pericarditis can be considered. These include infectious causes such as viral (including Coxsackie virus, adenovirus, and Epstein-Barr), bacterial (tuberculosis, Lyme disease, and Whipple’s disease), fungal, and parasitic. Neoplasm and paraneoplastic syndromes may cause pericardial effusions and/or pericarditis. Metabolic causes include hypothyroidism, uremia, and ovarian hyperstimulation syndrome. Radiation and trauma may also be potentiating factors in pericardial disease.

Treatment of pericarditis

Pericardial effusions in SLE are usually small and rarely compromise hemodynamics [7]. Retrospective reviews by Kahl et al. and Rosenbaum et al., however, each described 13% of pericarditis cases involving pericardial tamponade physiology [17-18].

Mild pericarditis in an SLE flare can be treated with intramuscular triamcinolone injection or oral methylprednisolone [32]. Severe pericarditis or pericardial tamponade should be treated with an intravenous bolus of methylprednisolone (initial dose usually 1 gram for three days) [7,16]. Tamponade with hemodynamic compromise should involve cardiology for emergent evaluation for pericardiocentesis, pericardial window, or pericardial stripping [16]. Intravenous immunoglobulin (IVIG), methotrexate, azathioprine, and mycophenolate mofetil have been described for recurrent pericarditis secondary to SLE [7, 16]. Colchicine has been demonstrated to reduce the risk of pericarditis recurrence in a meta-analysis [33]. Anakinra, an interleukin-1 inhibitor, has also been described as a safe and effective option in over 30 patients with recurrent steroid-dependent pericarditis unresponsive to conventional treatments, though has not been described in SLE patients [34-36].

## Conclusions

Pericarditis is a manifestation of SLE serositis recognized in the ACR, SLICC, and EULAR/ACR classification criteria of SLE. This is a common diagnosis in SLE, though alternative diagnoses should also be carefully considered in the evaluation.
